# A lightweight deep neural network for personalized detecting ventricular arrhythmias from a single-lead ECG device

**DOI:** 10.1371/journal.pdig.0001037

**Published:** 2025-10-08

**Authors:** Zhejun Sun, Wenrui Zhang, Yuxi Zhou, Shijia Geng, Deyun Zhang, Jiaze Wang, Bin Liu, Zhaoji Fu, Linlin Zheng, Chenyang Jiang, Guigang Zhang, Shenda Hong

**Affiliations:** 1 Department of Computer Science, Tianjin University of Technology, Tianjin, China; 2 National Institute of Health Data Science, Peking University, Beijing, China; 3 Institute of Medical Technology, Health Science Center of Peking University, Peking University, Beijing, China; 4 DCST, BNRist, RIIT, Institute of Internet Industry, Tsinghua University, Beijing, China; 5 HeartVoice Medical Technology, Hefei, China; 6 Department of Electrocardiography, The First Affiliated Hospital of Anhui Medical University, Hefei, China; 7 Department of Cardiology, Sir Run Run Shaw Hospital, Zhejiang University School of Medicine, Hangzhou, China; 8 Institute of Automation, Chinese Academy of Sciences, Beijing, China; Maastricht University Cardiovascular Research Institute Maastricht: Universiteit Maastricht Cardiovascular Research Institute Maastricht, NETHERLANDS, KINGDOM OF THE

## Abstract

Ventricular arrhythmia (VA) is a leading cause of sudden cardiac death. Detecting VA from electrocardiograms (ECGs) using deep learning techniques has potential to improve clinical outcomes. However, developing robust deep learning models for ECG analysis remains challenging due to: (1) inter-subject diversity among different individuals, and (2) intra-subject diversity within the same subject across different physiological state over time. In this study, we address these challenges by introducing enhancements in both the pre-training and fine-tuning stages. In the pre-training stage, we propose a novel approach combining model-agnostic meta-learning (MAML) with curriculum learning (CL) to effectively address inter-subject diversity. MAML efficiently transfer knowledge from large-scale datasets and enables rapid model adaptation to new individuals using limited records. Integrating CL further enhances the effectiveness of MAML by sequentially training models from simpler to more complex tasks. For the fine-tuning stage, we propose an improved pre-fine-tuning strategy specifically designed to manage the intra-subject diversity. We evaluate our methods on three publicly available ECG datasets and one real-world clinical ECG dataset collected using a portable device. Our proposed method achieves ROC-AUC = 0.984 / F1 = 0.940 with only 10 beats per class per subject on the test set and also achieves ROC-AUC = 0.965 / F1 = 0.937 on a real-world clinical collected data. Experimental results demonstrate that our proposed approach outperforms existing comparative methods across all evaluation metrics, and have a tendency to address intra-subject diversity. Ablation studies confirm that the combination of MAML and CL leads to more uniform performance across individuals, and our enhanced pre-fine-tuning technique substantially improves model adaptation to individual-specific data.

## Author summary

Ventricular arrhythmias are dangerous irregular heart rhythms that can lead to sudden cardiac death if they are not detected in time. Today, many people carry small, single-lead electrocardiogram (ECG) devices that record their heart signals at home or during daily activities. These devices create new opportunities for early warning, but existing computer algorithms often struggle to recognize abnormal rhythms in different people, or even in the same person as their condition changes.We developed a new lightweight deep learning system called MetaVA that learns to “adapt” to each individual quickly. Instead of needing thousands of training samples, the system can adjust itself with only a few labeled heartbeats from the user. We also designed a simple preparation step that helps the model stay accurate even as a person’s heart signals vary over time.In tests on large public databases and a clinical study with a handheld ECG device, our method reached high accuracy while requiring very little computing power. This means MetaVA could be embedded in wearable or portable devices, offering timely, personalized detection of life-threatening arrhythmias in everyday life.

## Introduction

Ventricular arrhythmias (VAs), such as ventricular tachycardia (VT) and ventricular fibrillation (VF), are the leading causes of sudden cardiac death worldwide [1]. These conditions significantly disrupt cardiac output, posing a high risk of sudden death. According to the American Heart Association (AHA), approximately 417,957 deaths in the United States in 2022 were attributed to cardiac arrest [2]. Early detection and timely intervention for out-of-hospital VAs have been shown to increase survival rates to 15 – 18 % survival [2].

Electrocardiography (ECG) is a noninvasive diagnostic tool that captures cardiac electrical activity, making it essential for the identification of abnormal ventricular patterns associated with VAs. In single-lead configurations, the ECG measures the potential difference along a single electrical axis defined by the electrode pair, thereby providing a projection of cardiac activity onto that axis. However, manual interpretation of ECG recordings by cardiologists is often time-consuming and prone to inconsistency [3,4].

Deep learning methods have shown promising results in ECG-based VA detection, and improve accuracy and consistency compared to manual interpretation and traditional machine learning approaches [5,6]. Notable examples include hierarchical-attention models that combine a dilated CNN front-end with dual (Bi-GRU + Bi-LSTM) recurrent streams [7], and ECG-to-image transformation techniques [8].

**Fig 1:**
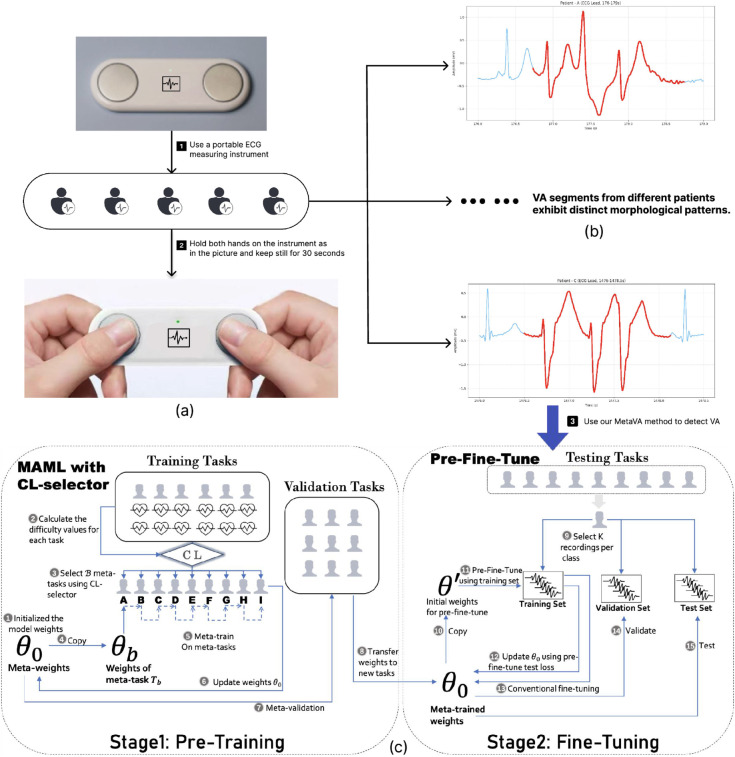
Overall workflow of our ventricular-arrhythmia detection system. **(a)** HeartVoice two–electrode recorder and fingertip-contact usage; **(b)** VA segments from different patients illustrating the large inter-patient morphology diversity that motivates personalised adaptation; **(c)** the proposed MetaVA pipeline: meta-trained on multi-subject tasks, then rapidly fine-tuned with a handful of beats from a target user for on-device inference.

Nevertheless, deep learning approaches still face two key challenges in VA detection:

- **Inter-subject diversity**: ECG signals differ among individuals due to differences in physiological characteristics and electrode placement, limiting model generalization [9]. For instance, VA segments from different patients exhibit distinct morphological patterns, as illustrated in Fig 1b.
- **Intra-subject diversity**: ECG signals from the same individual vary temporally due to physiological and activity changes, affecting model consistency [10].

To address these challenges, particularly inter-subject variability, we propose MetaVA, a meta-learning framework consisting of pre-training and fine-tuning stages. During pre-training, we integrate model-agnostic meta-learning (MAML) with curriculum learning (CL), enabling rapid adaptation to new individuals through an “easy-to-hard” learning sequence. Meta-learning provides a subject-agnostic initialization that quickly adapts to new individuals and shifting states, while curriculum learning stabilizes meta-optimization by pacing tasks from easy to hard across heterogeneous subjects. These properties target inter-subject variability and mitigate intra-subject drift. In fine-tuning stage, we employ a modified MAML-inspired approach to enhance robustness across diverse samples. Experimental results show that MetaVA outperforms existing methods, achieving improvements across all evaluation metrics.

## Related work

Traditional arrhythmia detection systems generally employ a two-stage approach: handcrafted features (e.g., RR intervals, QRS width/amplitude, wavelets) followed by classical classifiers such as SVM, K-NN, or decision trees [11]. These systems rely heavily on expert-selected features, making them fragile to ECG morphology variations and signal artifacts such as baseline drift and noise. As a result, these methods often generalize poorly to new subjects (inter-subject diversity) and tend to produce increasing false alarm rates in ambulatory settings [12].

End-to-end convolutional neural networks (CNNs) have been introduced to reduce manual feature engineering. For example, Acharya et al. proposed a 1-D CNN [13] and Mathews et al. developed a single-lead architecture [14] to learn hierarchical time–frequency representations directly from raw ECG signals. However, the existing high-performing models are heavily dependent on large, high-quality datasets [15]. Also, recent studies have pointed out that the fixed and limited receptive fields of CNNs struggle to capture the long-range temporal dependencies that are essential for discriminating complex arrhythmias [16]. Because rhythm patterns such as non-sustained ventricular tachycardia (NSVT—defined as runs of ≥3 consecutive ventricular beats, typically <30 s) and polymorphic VT, whose “twisting” QRS morphology evolves over several seconds, inherently require multi-beat temporal context.

To reduce data dependency, few-shot and personalized learning methods have been explored. Jia et al. fine-tune a large backbone network using only a few beats per patient, while meta-learning frameworks aim to accelerate this adaptation process [17]. Fan et al. further advance this direction by introducing knowledge-enhanced meta-transfer learning, achieving an F_1_ score of 92% from a single labeled beat through scaling and shifting a pre-trained backbone [18]. However, these approaches still rely heavily on extensive pre-training and the use of a 12-layer teacher model during distillation, leaving critical issues such as intra-subject stage shifts and device heterogeneity largely unexplored.

Hybrid CNN–Transformer models, such as MINA’s knowledge-guided attention network [19] and CAT-Net [20], have been developed to improve temporal sequence modeling in ECG analysis. While these models demonstrate enhanced representation capabilities, they introduce significant computational overhead, posing practical challenges for continuous monitoring on wearable devices. In addition, commonly used CNN backbones in ECG classification, such as Sakli et al.’s 12-lead ECG classifier based on ResNet-50 (≈25.6M parameters, ≈3.9 GFLOPs), underscore the model size burden [21]. Similar burdens are observed with pure-Transformer approaches; for instance, the recently proposed HeartBEiT instantiated a 12-layer ViT with ∼86M parameters and was pre-trained on 4×NVIDIA A100-40G GPUs for ∼2.5 months (300 epochs) on 8.5 million ECGs [22].

Lightweight CNN architectures, such as MobileNet and knowledge-distilled ECG models designed for embedded devices [23], aim to reduce model complexity through depth-wise separable convolutions, channel shuffling, and student–teacher compression. For instance, Chen et al. achieved a four-fold parameter reduction while preserving accuracy using a MobileNet-based classifier [24]. However, these compact models are rarely evaluated in low-resource, cross-patient scenarios with only a few available beats, and their actual effectiveness in reducing manual annotation effort remains largely unquantified.

Additionally, clinical ECG datasets are highly imbalanced, with normal beats vastly outnumbering pathological ones. To address this, recent studies have explored data augmentation techniques by generating minority-class samples. For example, HARDC uses a Conditional Generative Adversarial Network (CGAN) to synthesize ECG signals, combined with a class-balanced loss in its hierarchical attention RNN–dilated CNN architecture [7]. Another line of work generates synthetic beats using GANs and trains a lightweight Transformer model optimized with focal loss. The efficacy of such synthetic augmentation, especially under personalized learning setups, remains an open question [25].

To overcome these limits, we introduce MetaVA, a lightweight and robust VA detection framework coupled with MAML, curriculum task sampling, and a brief pre-fine-tuning step. MetaVA requires only a few labeled beats per subject, achieving generalization and computational efficiency suitable for wearable, low-resource settings.

## Method

As shown in Fig 1c, the overall MetaVA framework consists of two stages: MAML with CL-selector (pre-training, see Sect Pre-training) and pre-fine-tuning (fine-tuning, see Sect Pre-fine-tuning). The dataset includes multiple patients, each associated with several ECG segments. In this setup, each individual is treated as a meta task (details in Sect Pre-training), and the full task pool is divided into three task sets: training tasks, validation tasks and test tasks. During the pre-training stage, the model starts from randomly initialized weights $\theta_{0}$ and is trained across training tasks. The objective is to learn a generalizable initialization. Training continues until the validation loss on the validation tasks appears to increase, at which point the learned weights $\theta_{0}$ are considered optimal and transferred to the fine-tuning stage. In the fine-tuning stage, the pre-trained weights $\theta_{0}$ are individually adapted to each subject in the test set. This stage serves both as an adaptation step to unseen individuals and a final performance evaluation of the meta-initialization. Each test subject is fine-tuned separately and the adapted model is evaluated within that individual context. All key notations used in this study are summarized in Table 1.

**Table 1:** Notations.

Symbol	Description
𝒯	Set of meta-training tasks
𝒱	Set of difficulty values
${𝒯}_{train}$	Set of meta-training tasks
${𝒱}_{train}$	Set of difficulty values of ${𝒯}_{train}$
${𝒯}_{val}$	Set of meta-validation tasks
*T*_*b*_	A meta-task
$V_{b}$	The difficulty value of *T*_*b*_
*T*_*new*_	An unseen subject
$\theta_{0}$	Meta-weights
$\theta_{b}$	Copied parameters on meta-task *T*_*b*_
$f_{\theta }$	Model parameterized by *θ*
*D*_*s*_	Support set of a meta-task
*D*_*q*_	Query set of a meta-task
*D*_*t*_	The training set of *T*_*new*_
*K*	Number of segments selected per class
*B*	Number of meta-tasks for meta-training
ℬ	Size of a mini-batch of tasks

**Notes:** Definitions of symbols used throughout the manuscript.

### Pre-training

#### Meta-learning.

The field of meta-learning, also known as “learn-to-learn”, has gained increasing interest in recent years [26,27]. Meta-learning provides a method in which the trained model can gain experience over related tasks and improve its future learning performance using the experience [26]. MAML [28] is a widely used meta-learning method that learns a set of pre-trained weights over multiple tasks. As a model-agnostic meta-learning approach, MAML is designed to be independent of the underlying model architecture and learns a set of pre-trained weights over multiple tasks. Unlike traditional transfer learning, which attempts to find the global optimum across all pre-trained tasks, MAML focuses on the potential of the parameters by optimizing for rapid adaptability. Specifically, MAML aims to learn an initialization of model parameters that can quickly adapt to new target tasks with minimal updates, thereby achieving better performance on these tasks. Compared to other meta-learning methods, such as metric-based approaches that may require task-specific architectures, MAML’s model-agnostic nature allows it to generalize across diverse tasks without being constrained by the model structure or task-specific assumptions. In our application, we treat each person’s VA detection as a meta-task, leveraging the generalization power of MAML to quickly achieve VA detection for unseen patients.

Formally, we denote that the neural network $f_{\theta }$ is parameterized by *θ*. MAML attempts to find an initial set of parameters $\theta =\theta_{0}$ which could perform well on multiple unseen tasks after only several stochastic gradient descent steps. $\theta_{0}$, also called meta-weights, is not globally optimum on the pre-training tasks but has the greatest potential on unseen tasks—that is, to maximize the expected post-adaptation performance after *k* inner-loop steps on held-out subjects (e.g., ROC-AUC on the query set) relative to the pre-adaptation model. Therefore, $\theta_{0}$ is expected to be easily adaptable, which means it can be rapidly updated into a set of parameters that achieves good performance on a new task.

#### Curriculum learning.

Curriculum Learning (CL) [29] is a training strategy that encourages models to learn progressively from easy to difficult examples. This approach draws inspiration from human learning, where people are typically introduced to basic concepts before progressing to more complex ones. In our setting, we aim to organize meta-tasks in an “easy-to-hard” order. To do so, we first estimate the difficulty of each meta-task, defined as how difficult it is for the model to learn. Given a fixed model architecture and its initial weights, we can assign a constant value for each task as its “difficulty value”. By determining the values in advance, we can obtain an appropriate training order without introducing significant computational overhead.

For multi-task learning, we adopt a simple CL strategy. Assuming that the “difficulty values” of each task are $𝒱=\{V_{0},V_{1},\ldots ,V_{B}\}$ and the tasks are represented by 𝒯, we define a selection function sel(𝒯,𝒱,i) to calculate the selection probabilities at the i-th iteration. Based on these probabilities, a roulette wheel selection is performed to sample a mini-batch of tasks.

#### MAML with CL-selector.

We aim to employ MAML to achieve a rapid adaptation of VA detection to new individuals. We regard VA detection of each subject as a meta-task, and our target is to pre-train the model across multiple meta-tasks, and then transfer the pre-trained weights to unseen patients so that we can use several segments of the unseen patient to fine-tune the pre-trained model and achieve better performance.

During pre-training (also referred to as *meta-train*), the model is trained on a large-scale VA dataset collected from *B* patients, represented as $𝒯=\{T_{1},T_{2},\ldots ,T_{B}\}$, where *T*_*i*_ is the i-th meta-task. This stage yields a meta-trained parameter set $\theta_{0}$, which serves as the initialization point for future adaptions. In each epoch of meta-training, we employ a task-selector (referred to as CL-selector) to determine which meta-tasks to use in the current mini-batch. We consider that $\theta_{0}$ consolidates the knowledge of all the meta-tasks. When faced with a new unseen task *T*_*new*_, we can transfer the pre-trained parameters $\theta_{0}$ to *T*_*new*_. The details of pre-training are described as follows.

We divide all meta-tasks in pre-training stage 𝒯 into two sets: training tasks set (${𝒯}_{train}$) and validation tasks set (${𝒯}_{val}$). Each subject in the dataset is treated as a single meta-task (*T*_*b*_). The model weights are initially assigned using Xavier initialization [30], which is a widely used randomized weight initialization method.

As described in Table 2 and Fig 1c, the difficulty values for each task are first calculated to obtain ${𝒱}_{train}$. Specifically, the model with weights $\theta_{0}$ is trained using *K* records per class in each meta-task $T_{b}\in {𝒯}_{train}$ and evaluated on the remaining records of *T*_*b*_. For each *T*_*b*_, the average loss *loss*_*b*_ is obtained across all test records. The corresponding difficulty value $V_{b}$ is then computed by applying the softmax function to the set of average losses:

$$V_{b}=Softmax(loss_{b})=\frac{e^{loss_{b}}}{\sum_{i=1}^{B}e^{loss_{i}}}.$$ (1)

**Table 2:** Algorithm: MAML with CL-selector.

**Input:** Meta-training tasks ${𝒯}_{train}$ **Output:** Optimized meta-weights $\theta_{0}$
1. Obtain difficulty values ${𝒱}_{train}$ using CL-selector initialization (Table 3).
2. For each meta-training iteration *iter*:
a. Select a mini-batch of tasks: ${𝒯}_{batch}$ = CL-selector(${𝒯}_{train}$, ${𝒱}_{train}$, *iter*, ℬ) (Table 4).
b. For each meta-task $T_{b}\in {𝒯}_{batch}$:
i. Initialize task weights: $\theta_{b}\leftarrow \theta_{0}$.
ii. Randomly select *K* epochs per class for support set *D*_*s*_ and query set *D*_*q*_.
iii. For step=1→updates:
- Compute loss on *D*_*s*_: ${ℒ}_{T_{b}}^{D_{s}}(f_{\theta_{b}})$.
- Update $\theta_{b}$ using Eq 5.
- Compute loss on *D*_*q*_: ${ℒ}_{T_{b}}^{D_{q}}(f_{\theta_{b}})$.
- Append ${ℒ}_{T_{b}}^{D_{q}}$ to ${𝕃}_{T_{b}}$.
c. Compute the total meta-loss across tasks using Eq 6.
d. Update meta-weights $\theta_{0}$ using Eq 7.
3. Return optimized $\theta_{0}$.

**Notes:** This algorithm integrates curriculum learning into MAML, ensuring task sampling progresses from easy to hard, while meta-optimization yields a generalizable initialization $\theta_{0}$.

After the difficulty values are calculated, the pre-training stage (meta-training) is initiated. During each meta-training iteration, a mini-batch of ℬ meta-tasks is selected using the CL-selector. The CL-selector integrates a selection function sel(·), which depends on the current iteration *iter*, the number of times a task *T*_*k*_ has been selected (*t*_*k*_), and its difficulty value $V_{k}$. The probability of selecting each meta-task is determined by this function, followed by roulette-wheel sampling. The selection function is defined as:

$$sel(iter,t_{k},V_{k})=\frac{\max(0,\;𝕀(V_{k}<thres)-\frac{t_{k}}{iter})}{\sum_{k=1}^{B}𝕀(V_{k}<thres)},$$ (2)

where the threshold is given by

$$thres=\max(e^{\;iter-MaxIter},\;\text{lowest}),$$ (3)

and 𝕀(·) denotes the indicator function:

$$𝕀(x<a)=\left\{ \begin{array}{cc} 1, & x<a,\\ 0, & x\geq a. \end{array}\right.$$ (4)

Here, *lowest* denotes the ℬ-th smallest difficulty value, and *MaxIter* is set to 50, representing the iteration after which all tasks are eligible for selection. The use of softmax converts the average losses into a probability distribution over tasks. During task selection, a threshold relative to *iter* is applied, and tasks with difficulty values below this threshold are chosen. To further balance training, the probability of repeatedly selecting previously used tasks is reduced. The overall process for the CL-selector is summarized in Tables 3 and 4.

**Table 3:** Algorithm: CL-selector initialization.

**Input:** Training task set ${𝒯}_{train}$ **Output:** Difficulty values ${𝒱}_{train}$
1. For each meta-task *T*_*b*_ in ${𝒯}_{train}$:
a. Train the model using *K* records of *T*_*b*_.
b. Calculate the loss *loss*_*b*_ on the other recordings of *T*_*b*_.
c. Compute the average loss: $loss_{b}\leftarrow \frac{loss_{b}}{|T_{b}|-K}$.
d. Save *loss*_*b*_ in ${𝕃}_{loss}$.
2. Apply *Softmax* on ${𝕃}_{loss}$ using Eq 1 to obtain ${𝒱}_{train}$.
3. Return ${𝒱}_{train}$.

**Notes:** This algorithm estimates difficulty values for curriculum task sampling. These values are later used in the CL-selector to schedule tasks from easy to hard.

**Table 4:** Algorithm: CL-selector.

**Input:** Training tasks ${𝒯}_{train}$, difficulty values ${𝒱}_{train}$, current iteration *iter*, batch size ℬ, maximum iteration *MaxIter*, lowest threshold *lowest* **Output:** Selected task indices 𝕋
1. Compute threshold: $thres=V_{k}<\max(e^{iter-MaxIter},lowest)$.
2. For b=1→ℬ:
a. For each meta-task *T*_*b*_ in ${𝒯}_{train}$:
i. Calculate the probability *P*_*b*_ of selecting *T*_*b*_ using Eq 2.
ii. Retain cumulative probability $P_{b}+P_{b-1}$ (or *P*_*b*_ if *b* = 1) in ℙ.
b. Generate a random number rand∈[0,1].
c. Find index *t* where rand≤ℙ[t].
d. Save *t* in 𝕋.
3. Return 𝕋.

**Notes:** The CL-selector samples meta-tasks based on difficulty values, gradually shifting from easier to harder tasks, ensuring curriculum-style task scheduling.

After selecting a mini-batch, the model is meta-trained using all tasks in this mini-batch. Subsequently, the support set *D*_*s*_ and query set *D*_*q*_ are randomly selected from *T*_*b*_, and each of these consists of *K* records for VA and non-VA respectively. Thus, a total of K×2 records are selected per set. Then, we perform gradient descent separately for *update* steps. For each *T*_*b*_, we firstly copy the current weights $\theta_{0}$ as $\theta_{b}$ to ensure the same start weights. Then we calculate the loss on *D*_*s*_ (${ℒ}_{T_{b}}^{D_{s}}$) and update $\theta_{b}$ as follows:

$$\theta_{b}\leftarrow \theta_{b}-\alpha \nabla_{\theta_{b}}{ℒ}_{T_{b}}^{D_{s}}(f_{\theta_{b}})$$ (5)

where *α* is the learning rate (we call it *update_lr* to distinguish the following *meta_lr*). Next, we evaluate the updated weights $\theta_{b}$ on the query set *D*_*q*_, and obtain the loss on *D*_*q*_ (${ℒ}_{T_{b}}^{D_{q}}$) which is retained in a list ${𝕃}_{T_{b}}$. After all ℬ meta-tasks selected are completed, we obtain the summation of losses on ℬ query sets:

$${ℒ}_{meta}(\theta_{0})=\sum_{b=1}^{ℬ}{𝕃}_{T_{b}}(f_{\theta_{b}})$$ (6)

This is actually the objective function for meta-training, and our goal is to minimize it. It is to be observed that the loss is a function of $\theta_{0}$ rather than $\theta_{b}$. To update $\theta_{0}$, we perform gradient descent of ${ℒ}_{meta}(\theta_{0})$ using

$$\theta_{0}\leftarrow \theta_{0}-\gamma \nabla_{\theta_{0}}{ℒ}_{meta}(\theta_{0})$$ (7)

where *γ* is the learning rate of meta-training (i.e., *meta_lr* as mentioned earlier).

In the meta-validation stage, we adapt the model to all meta-tasks in ${𝒯}_{val}$ and test the performance. In detail, we transfer the pre-trained $\theta_{0}$ to each subject of validation tasks set. For each individual, all segments are divided into training set and test set, and we train the model using the training set and evaluate the model on the test set. The validation loss of the individual is the sum of the losses on the test set for this person, and the total validation loss is the sum of validation losses of all individuals. This procedure is used to tune the hyperparameters such as learning rates and *updates*. When obtaining the lowest overall validation loss for all the new validation meta-tasks, we keep $\theta_{0}$ for the further fine-tuning step.

### Pre-fine-tuning

This stage personalizes fine-tuning for each new target individual. Instead of directly applying traditional transfer learning methods, we introduce a MAML-inspired “pre-fine-tuning” step before standard fine-tuning. Pre-fine-tuning brings meta-learning logic into the fine-tuning stage. This addresses variability in ECG recordings from the same person over time, bridging the gap between pre-training and fine-tuning.

In MAML, the model minimizes the loss of updated weights $\theta_{b}$ (copied from meta-weights $\theta_{0}$ and fine-tuned on a task’s support set) using a “future” loss for adaptability. Similarly, for unseen individuals, we apply a “future” loss via pre-fine-tuning, followed by standard fine-tuning with the “current” loss. This reduces the discrepancy between meta-learning and direct fine-tuning.

We initialize the model with pre-trained weights $\theta_{0}$ and split the unseen subject’s records ($T_{\text{new}}$) into a training set (*D*_*t*_) with 2*K* records (*K* per class), a validation set with 2*K* records (*K* per class), and a test set with the remaining records.

In pre-fine-tuning (Table 5), we copy $\theta^{\prime }$ from $\theta_{0}$, then update $\theta^{\prime }$ to $\theta^{\prime \prime }$ using *D*_*t*_:

$$\theta^{\prime \prime }\leftarrow \theta^{\prime }-\beta_{1}\nabla_{\theta^{\prime }}{ℒ}_{D_{t}}(f_{\theta^{\prime }})$$ (8)

where $\beta_{1}$ is the learning rate. Next, we compute the loss ${ℒ}_{D_{t}}(f_{\theta^{\prime \prime }})$ and update $\theta_{0}$:

$$\theta_{0}\leftarrow \theta^{\prime \prime }-\beta_{2}\nabla_{\theta^{\prime \prime }}{ℒ}_{D_{t}}(f_{\theta^{\prime \prime }})$$ (9)

with learning rate $\beta_{2}$.

**Table 5:** Algorithm: Pre-fine-tuning.

**Input:** Training set *D*_*t*_ **Output:** Updated weights $\theta_{0}$
1. For each pre-fine-tuning iteration:
a. Copy the pre-trained weights: $\theta^{\prime }\leftarrow \theta_{0}$
b. Calculate loss of $\theta^{\prime }$: ${ℒ}_{D_{t}}(f_{\theta^{\prime }})$
c. Update weights to obtain $\theta^{\prime \prime }$ using Eq 8
d. Calculate loss of $\theta^{\prime \prime }$: ${ℒ}_{D_{t}}(f_{\theta^{\prime \prime }})$
e. Update $\theta_{0}$ using Eq 9

**Notes:** This procedure introduces a MAML-inspired pre-fine-tuning step, bridging meta-learning initialization with personalized adaptation.

After pre-fine-tuning, traditional fine-tuning applies stochastic gradient descent on *D*_*t*_ to refine the weights. With a small *K*, the model adapts efficiently to new targets. Validation tunes hyperparameters, and testing evaluates final performance.

### Implementation details

In this section, we describe the implementation details of MetaVA. For a better understanding, we follow the basic pipeline in Fig 1c and describe pre-training and fine-tuning separately. The experimental setup for these processes is also detailed to provide context for the implementation.

#### Architecture of deep neural network.

Our method is model-agnostic and does not require any specific form of deep neural network. Therefore, the purpose of the experiments is not to compare different network architectures. We use one lightweight neural network architecture throughout the experiments.

The selected model is modified based on a deep neural network backbone that achieves state-of-the-art performance in ECG modeling [31]. It can be seen as a structurally designed [32] one-dimensional ResNeXt [33] model, with several layers removed to achieve a lightweight architecture and the detailed model architecture is shown in Table 6. The network consists of 8 layers, processing data through a series of convolutional, normalization, pooling, and transformer operations. It begins with two parallel convolutional layers, Conv1 and Conv2, using 1D convolutions with kernel sizes of 5 and 9, stride of 1, and padding of 2 and 4, respectively. Their outputs are fused via stacking and averaging. The fused features undergo batch normalization, ReLU activation, max pooling with kernel size 2, and dropout, halving the sequence length. Conv Block1 follows, featuring a 1D convolution with kernel size 8, padding 4, batch normalization, ReLU, and adaptive average pooling to a fixed length of 50, expanding the feature depth to 64. The data is then permuted and processed by a single Transformer Encoder Layer [34]. Finally, global average pooling reduces the output to 64, and a fully connected layer to do binary classification.

**Table 6:** Our network architecture.

Layer Name	Composition	Output Size
Conv1	Conv1d(1, 128, kernel_size=5, stride=2, padding=2)	[${\;\;}^{*}$, 128, 400]
Conv2	Conv1d(1, 128, kernel_size=9, stride=2, padding=4)	[${\;\;}^{*}$, 128, 400]
Feature Fusion	torch.stack + mean(dim=2)	[${\;\;}^{*}$, 128, 400]
BN + ReLU + MP + DO	BatchNorm1d(128), ReLU, MaxPool1d(2), Dropout(0.5)	[${\;\;}^{*}$, 128, 200]
Conv Block1	Conv1d(128, 64, kernel_size=8, padding=4), BN, ReLU, AdaptiveAvgPool1d(50)	[${\;\;}^{*}$, 64, 50]
Permute	permute(0, 2, 1)	[${\;\;}^{*}$, 50, 64]
Transformer Encoder	TransformerEncoderLayer(d_model=64, nhead=8)	[${\;\;}^{*}$, 50, 64]
Global Avg Pool	mean(dim=1)	[${\;\;}^{*}$, 64]
Classifier	Linear(64, 2)	[${\;\;}^{*}$, 2]

In addition, we profiled on-device efficiency at the segment (window) level used for evaluation. This lightweight network contains only 0.596M parameters (2.21 MB in FP32) and requires approximately 17.4 MFLOPs per window for typical input lengths (L=400–2880 samples), achieving 0.40–0.73 ms single-thread CPU latency (batch = 1, eval/no_grad).

#### Pre-training.

As described in Sect MAML with CL-selector, ℬ meta-tasks are selected by the CL-selector in each meta-training iteration, and *K* records for each class are randomly selected for training in each meta-task *T*_*b*_ (i.e., each subject). To achieve fast adaptation, the value of *K* should not be too large and is set as 10. The number of meta-tasks selected per iteration (ℬ) is 9. Meta-training continues until the meta-validation loss shows an increasing trend, at which point the best weights $\theta_{0}$ are saved. Hyperparameters include: updating learning rate *update_lr* (*α*) $\in \{10^{-2},5\;\times \;10^{-3},10^{-3}\}$, meta-learning rate *meta_lr* (*γ*) $\in \{10^{-2},5\times 10^{-3},10^{-3}\}$, and the number of updating steps (updates∈{1,3,5}).

#### Fine-tuning.

The pre-trained weights $\theta_{0}$ are transferred to the second stage to adapt quickly to new subjects. Fine-tuning involves a pre-fine-tuning step followed by traditional fine-tuning, as described in Sect Pre-fine-tuning. After pre-fine-tuning, gradient descent is applied until the training loss stabilizes. The fine-tuned model is then tested on the remaining recordings. To avoid any leakage from overlapping windows, we adopt a temporal-disjoint protocol at the subject level. For each held-out subject, we first select the support set for adaptation (*K* segments per class; K=10 unless otherwise stated) and mask out all windows that overlap with these support windows or fall within a safety gap equal to one window length on either side. From the remaining non-overlapping windows we draw a validation subset under the same non-overlap constraint, and designate the rest as the test set. This guarantees that no temporally adjacent (near-duplicate) segments appear across support/validation/test for a given subject. And the hyperparameters include: the number of pre-fine-tuning iterations (*iter* ∈{10,30,50}), two learning rates in pre-fine-tuning ($\beta_{1},\;\beta_{2}\in \{10^{-2},5\times 10^{-3},10^{-3},5\times 10^{-4}\}$), and the learning rate in fine-tuning (*learning_rate*$\in \{10^{-2},10^{-3},10^{-4}\}$). The number of updating steps (updates∈{1,3,5}) and the maximum number of training iterations in fine-tuning is set to 300.

#### Experimental setup.

For pre-training, we combine MITDB and CUDB to expose the model to complementary VA patterns at the subject level: MITDB offers a broad spectrum of annotated beats across many individuals, while CUDB contributes sustained VT/VF episodes that reflect clinically salient, longer VA runs. To evaluate cross-dataset generalization and to prevent information leakage, we reserve VFDB exclusively for meta-validation and model selection, with no subject overlap with meta-training. This design intentionally creates a distributional shift between pre-training (MITDB+CUDB) and fine-tuning (VFDB), which aligns with our goal of robust adaptation to unseen subjects and cohorts. To ensure robustness, the model is trained five times with the best hyperparameters, and each fine-tuning configuration is run 10 times per subject.

## Results

To verify the feasibility of using MAML, the CL-selector, and pre-fine-tuning for VA detection, we conduct two-part experiments with pre-training and fine-tuning. Our hypotheses are: (1) MAML produces better initial weights compared to traditional pre-training methods; (2) the CL-selector can help MAML find more suitable parameters; (3) pre-fine-tuning can improve the performance of traditional fine-tuning when we adapt the model to new people.

### Datasets

In the experiments, we use the following three publicly available and one single-lead real-world ECG datasets:

- MIT-BIH arrhythmia database (MITDB) [35]. MITDB was set up by the Beth Israel Hospital Arrhythmia Laboratory between 1975 and 1979, containing 48 30-minute recordings obtained from 47 subjects. Each ECG recording has two channels (leads) and the sampling rate is 360 Hz. Twenty-three recordings were randomly selected and the other 25 recordings represented less common but clinically important phenomena. This database is the first publicly available data set to evaluate arrhythmia detectors.
- MIT-BIH malignant ventricular ectopy database (VFDB) [36]. VFDB was collected from the ECG tape libraries of the Brigham and Women’s Hospital and Beth Israel Hospital in Boston in 1986, containing 22 half-hour recordings obtained from 16 patients. Each recording has two channels as well, and the sampling rate is 250 Hz. This database is widely used in VA detection tasks.
- Creighton University ventricular tachyarrhythmia database (CUDB) [37]. CUDB was originally collected by Floyd M. Nolle at the Creighton University Cardiac Center. It contains 35 eight-minute ECG recordings with sustained VT, VF, and ventricular flutter. All signals are digitized at 250 Hz with 12-bit resolution, and each recording has only one channel. The high-quality VA-specific recordings are beneficial for VA detection tasks.
- HeartVoice VT Clinical Data [38]. To evaluate the real-world applicability of MetaVA and demonstrate its algorithmic improvements, we utilized continuous single-lead ECGs from 30 VT patients collected via the HeartVoice platform (https://www.heartvoice.com.cn). Unlike public recordings, which are often dated and acquired under relatively controlled or resting conditions, HeartVoice data are obtained through modern ambulatory monitoring in real clinical settings. These up-to-date recordings capture a broader range of patient activity and physiological variability, making them more suitable for modeling the diverse ECG patterns encountered in practical deployment scenarios.

A summary of these databases is presented in Table 7.

**Table 7:** Summary of databases.

Database	Recordings	Leads	Rate (Hz)	Duration (min)	VA:non-VA (segment-level)	VA:non-VA (orig. duration)
*Public Datasets*						
MITDB	48	2	360	30	146:42536 (0.34%)	357:86310 (0.4%)
VFDB	22	2	250	30	5965:20208 (22.7%)	7629:38571 (16.5%)
CUDB	35	1	250	8	6129:7255 (45.8%)	3841:13972 (21.6%)
*Real-world collected dataset*						
HeartVoice VT Data	30	1	125	3	3500:5566 (38.6%)	900:4500 (16.7%)

**Notes:** Ratios in the “segment-level” column are computed **after windowing** (window = 2 s). The “orig. duration” column is computed **before windowing**, based on raw recording time.

On the ECG datasets, we apply the following pre-processing steps: (1) For MITDB, we use the upper channel (MLII), as recommended in the database documentation and widely adopted in prior work due to its prominent QRS morphology. For VFDB, since header files typically list channels generically as “ECG” without a specific lead label, we follow common practice and use the first channel. And we use the unique channel from CUDB and Heart Voice VT data. (2) All recordings are re-sampled to 200 Hz using linear interpolation. (3) Long recordings are split into 2-second segments (records) using a sliding window with dynamic strides. (4) Each segment is labeled as 1 (if VA exists) or 0 (if not). In addition, to deal with the imbalance problem, the stride is set to 20 (0.1 s × 200 Hz) if VA exists and 400 (2 s × 200 Hz) if not. Sect Implementation details provides more information on the partitioning of training, validation and test sets as well as method details.

The performance of MetaVA and the comparison methods is evaluated using the following metrics: receiver operating characteristics area under the curve (ROC-AUC), precision-recall area under the curve (PR-AUC), accuracy (ACC), and F1-score. The ROC-AUC can measure a binary classifier system as the discrimination threshold varies. It can also be regarded as the probability that a positive example ranks higher than a negative example when randomly chosen. PR-AUC represents the mean precision under different discrimination thresholds. F1-score is the harmonic mean of the precision and recall $F_{1}=2\;\times \;\frac{precision\times recall}{precision+recall}$. Accuracy is the ratio of the number of truly classified samples to the number of samples. For the last two metrics, we obtain the threshold for classifying labels by maximizing the geometric mean value.

### Performance of MetaVA

The MetaVA model integrates Model-Agnostic Meta-Learning (MAML) with a curriculum learning (CL) selector during pre-training and a pre-fine-tuning strategy for subject-specific adaptation. It achieves state-of-the-art performance in ventricular arrhythmias (VA) detection, with an ROC-AUC of 0.9843 (95% CI: 0.9705–0.9916), accuracy of 0.9602 (95% CI: 0.9176–0.9635), balanced accuracy 0.9477, PR-AUC of 0.9681 (95% CI: 0.9607–0.9914), and F1-score of 0.9401 (95% CI: 0.9042–0.9599), where confidence intervals were obtained via subject-level bootstrap (20,000 resamples). As shown in Table 9, these results demonstrate the model’s robust ability to distinguish VA from non-VA cases while maintaining high precision and recall. The performance highlights MetaVA’s capability to rapidly adapt to new subjects with limited data, making it a highly effective tool for VA detection in clinical settings where both accuracy and adaptability are critical.

### Comparison methods

To assess the performance of MetaVA, we compare it with the following state-of-the-art methods for ventricular arrhythmia (VA) detection:

- **ECGTransForm** [31]: This framework introduces a Bidirectional Transformer (BiTrans) to capture both past and future temporal dependencies in ECG signals. It employs Multi-scale Convolutions (MSC) to extract spatial features across varying granularities and a Channel Recalibration Module (CRM) to enhance cross-channel feature interactions. And the lightweight model which MetaVA uses is an adaptation of this.
- **Metric-based Meta-learning** [39]: This approach tackles limited abnormal ECG samples using a metric-based meta-learning framework. The Meta-Siamese Network (MSN), integrating Siamese and residual networks, learns similarity metrics between sample pairs, enabling effective few-shot classification.
- **LightX3ECG** [40]: This lightweight system uses three separate 1D-SEResNet18 backbones to process each lead independently, optimizing feature extraction. A Lead-wise Attention module aggregates these features, identifying the most influential lead, while Lead-wise Grad-CAM generates per-lead class activation maps, enhancing interpretability.

These methods highlight advancements in temporal modeling, interpretability, and few-shot learning, critical for arrhythmia detection. In order to use these methods, the final output is changed from multi-classification to VA detection of binary classification, ensuring the original model architecture and algorithm.

The results, summarized in Table 9, demonstrate that MetaVA consistently outperforms existing approaches across all assessed metrics. MetaVA achieves the highest values in both ROC-AUC and ACC, reflecting its superior class discrimination and overall predictive reliability. It also shows competitive results in PR-AUC, F1-score, recall, and precision. Notably, MetaVA improves upon ECGTransform by 1.44% in ROC-AUC and 2.76% in ACC, and surpasses Metric-Based MetaLearning by 3.88% in ROC-AUC and 2.53% in ACC. The model’s strong performance in ROC-AUC and PR-AUC underscores its robustness in handling imbalanced datasets, which is essential for VA detection.

### Ablation study

#### Contributions of the key components.

**Fig 2:**
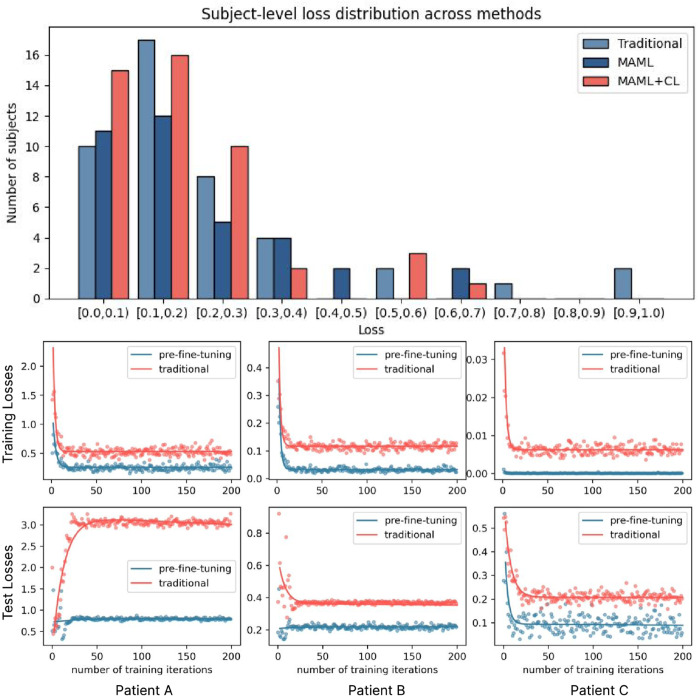
Loss–profile comparison analysis for MetaVA. **Top panel**: distribution of validation loss values after pre-training across all source subjects—each bar counts the number of subjects. **Bottom panel**: detailed learning curves for the three most difficult target subjects.

To evaluate the contributions of the key components in MetaVA, we compare the full MetaVA model with several variants:

- **MAML without CL-selector (MAML).** During pre-training, meta-tasks are selected randomly (no CL-selector) to test the effect of curriculum learning; all other hyperparameters remain the same. Under direct fine-tuning, MAML-initialized weights outperform traditional direct pre-training (ROC-AUC: 0.9678 vs. 0.9566; F1: 0.8932 vs. 0.8833), attributable to MAML’s more balanced subject-level generalization compared with the uneven loss distribution in direct pre-training (Fig 2). A paired t-test confirms significance (p-value: $1.02\;\times \;10^{-7}$). Moreover, adding the CL-selector on top of MAML further improves performance (PR-AUC: 0.9443 vs. 0.9426 under direct fine-tuning; 0.9681 vs. 0.9588 with pre-fine-tuning) and yields a more balanced loss distribution (Fig 2); one-sided t-test shows statistical significance (p-value = 0.033).
- **Direct pre-training.** To match the sample count used by our meta-training (which requires two sets *D*_*q*_ and *D*_*s*_), we select ℬ×K×2 records per class for each subject in every iteration. Hyperparameters: learning rate $\in \{10^{-2},5\;\times \;10^{-3},10^{-3}\}$ and batch size ∈{32,64,128}. As a baseline, direct pre-training achieves ROC-AUC 0.9566 and F1 0.8833 under direct fine-tuning, which is lower than with MAML initialization; it also exhibits a more uneven loss distribution (Fig 2).
- **Direct fine-tuning.** Used to verify whether *pre-fine-tuning* improves over direct fine-tuning; the only hyperparameter is *learning_rate* $\in \{10^{-2},10^{-3},10^{-4}\}$, with a maximum of 200 training iterations. Relative to this direct fine-tuning control, pre-fine-tuning provides consistent gains across key metrics—for example, ROC-AUC increases by 1.01% to approximately 0.9843, with similar improvements in PR-AUC and F1—and yields more stable training under challenging conditions, with smoother training and test loss curves (Fig 2).

These variants are combined in different ways (e.g., MAML with direct fine-tuning, MAML with CL-selector and direct fine-tuning) to isolate the effects of each component. The combinations are summarized in Table 8, and their performances are compared in Table 9. Notice that MC+F is an advanced edition of MetaSleepLearner [41], from which our MAML approach is inspired.

**Table 8:** The combinations represented by compared methods and ours.

	Pre-Training Stage	Fine-Tuning Stage
MetaVA	MAML with CL-selector	Pre-Fine-Tune
MC+F	MAML with CL-selector	Fine-Tune
M+PF	MAML	Pre-Fine-Tune
M+F	MAML	Fine-Tune
Vanilla	Pre-Training	Fine-Tune

**Table 9:** Overall results: (top) ablation combinations of MetaVA, (bottom) comparison with state-of-the-art methods. Boldface marks the best score.

Method	ROC-AUC	Accuracy	PR-AUC	F1-score
*Ablation study on MetaVA*				
Our Proposed Method MetaVA	0.9843	0.9602	0.9681	0.9401
MC+F	0.9733	0.9345	0.9443	0.9227
M+PF	0.9705	0.9142	0.9442	0.8961
M+F	0.9678	0.9105	0.9426	0.8932
Vanilla	0.9566	0.9392	0.9025	0.8833
*External comparison methods*				
Metric-Based Meta-Learning	0.9438	0.9238	0.9341	0.9177
ECGTransForm	0.9682	0.9216	0.9625	0.9081
LightX3ECG	0.9687	0.9290	0.9582	0.9184

#### Periodic curriculum refresh of task-difficulty scores.

To evaluate whether dynamically updating task-difficulty scores benefits learning, we implemented a periodically refreshed curriculum and compared it to the frozen-score baseline. On our setup, a full refresh over all training tasks takes approximately ∼1 minute. We tested refresh schedules every {20, 50, 100} epochs; for a standard run, this adds roughly {15, 6, 3} minutes of wall-clock time, respectively.

Across datasets, no material gains were observed: post-adaptation ROC-AUC and F1 on held-out subjects remained within ±0.1–0.2 percentage points of the frozen baseline, and in some runs the refreshed curriculum was slightly worse. Given the extra wall-clock overhead without accuracy benefits, we retain the frozen-score design in our main pipeline.

#### Backbone-agnostic evaluation protocol.

To assess whether the proposed MetaVA (MAML + CL-selector + pre-fine-tuning) is tied to a particular feature extractor, we evaluate it on **three representative 1D temporal encoders** in addition to our main CNN–Transformer backbone (Table 6):

- **Pure CNN (Net1D):** a convolution-only backbone with 1D convolutions and global pooling (no recurrence or attention).
- **CNN+RNN (HARDC-like):** a hybrid architecture that combines a dilated-CNN front-end with a bidirectional recurrent layer (BiGRU/BiLSTM), following the design spirit of HARDC [7].
- **Pure Transformer (ViT-style):** a vision-transformer–style encoder adapted to 1D ECG segments (patch embedding + stacked MHSA blocks + MLP head), inspired by [16].

All backbones are implemented in the same way. We report subject-level metrics including ROC-AUC, PR-AUC, F1, accuracy (ACC), and balanced accuracy (BACC). Table 10 summarizes MetaVA’s subject-level performance when instantiated with different backbones. Across all three backbones, adding MetaVA (MAML + CL-selector + pre-fine-tuning) produced consistent gains over baselines, while the absolute performance levels were comparable to those reported with our original lightweight backbone. These observations indicate that the benefits of MetaVA are not tied to a specific feature extractor and persist across disparate encoder families.

**Table 10:** Performance of MetaVA across different backbones. All runs follow the identical meta-training / meta-validation / fine-tuning protocol.

Architecture	AUC	ACC	BACC	PR-AUC	F1
Pure CNN — Net1D	0.9810	0.9551	0.9567	0.9702	0.9512
CNN+RNN — HARDC-like [7]	0.9432	0.9232	0.9089	0.9235	0.9129
Pure Transformer — ViT-style [16]	0.9716	0.9248	0.9279	0.9496	0.9138
CNN+Transformer — (main backbone)	0.9843	0.9602	0.9477	0.9681	0.9401

### Backbone capacity and compatibility

We analyzed how MetaVA behaves across backbones with different capacities and inductive biases. Concretely, we profiled four representative 1D temporal encoders (pure CNN: Net1D; CNN+RNN: HARDC-like; pure Transformer: ViT-style; CNN+Transformer: our main backbone) under the same input window and batch size. Table 11 summarizes efficiency indicators (parameters, FLOPs per segment, and CPU (1-thread) latency), while Table 10 reports subject-level macro performance.

**Table 11:** Backbone efficiency profiling (same input window, batch=1, CPU 1-thread).

Backbone	Params (M)	FLOPs (MFLOPs)	Latency (ms)
Pure CNN — Net1D	0.358	100.76	2.962
CNN+RNN — HARDC-like	0.847	1620.00	74.942
Pure Transformer — ViT	0.801	38.24	0.833
CNN+Transformer — (ours)	0.596	17.44	0.482

Overall, MetaVA attains strong performance on lightweight models (sub-million parameters, sub-millisecond CPU latency), and we do not observe a monotonic relationship between model size and downstream VA detection metrics. Notably, the CNN+Transformer backbone used in our main experiments combines one of the smallest FLOPs with the best AUC, while the highest-parameter/highest-FLOPs hybrid RNN is both slower and weaker under few-shot personalization. These results support our claim that the method is compatible with lightweight backbones suitable for wearable deployment.

### Robustness to limited per-subject volume

To probe the “limited-data individual” setting, we re-tested on the HeartVoice cohort (shorter recordings) with adaptation budgets K={5,10,20} segments per class and used the remaining windows for testing. Using K=5 simulates very scarce per-subject data and led to less stable and noticeably worse outcomes; when using K=10 or 20, the results were essentially the same as those reported.

Importantly, even at small budgets the method remained usable for most subjects: with K=5, approximately 70% of patients still achieved AUC ≥0.95 and F1 ≥0.95. Cohort-level macro-averages for K=5 were: AUC=0.914,  ACC=0.883,  PR-AUC=0.883,  F1=0.907.

(For K=10/20, metrics were essentially unchanged from those already reported.)

### Performance under extreme class imbalance

We quantified the VA:non-VA imbalance ratios in Table 7. To examine performance under extreme prevalence, we analyzed representative VFDB patients with VA:non-VA prevalences around 2%, 10%, and 40%, and report their per-subject metrics below. As expected, in the most extreme low-prevalence case (2% VA) the performance is less stable and somewhat lower than at milder prevalences; 10%/40% cases are comparatively stronger (Table 12).

**Table 12:** Representative VFDB subjects under different VA prevalence levels.

VA : non-VA	AUC	ACC	PR-AUC	F1
2%	0.937	0.924	0.821	0.903
10%	0.982	0.945	0.987	0.962
40%	0.999	0.985	0.998	0.985

### Real world validation on heartVoice clinical VT data

**Fig 3:**
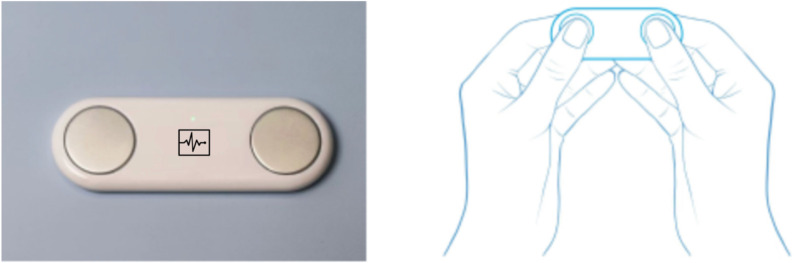
Portable handheld recorder and typical two-finger grip used by patients during ECG acquisition.

Fig 3 illustrates the HeartVoice sensor and its two-finger grip ECG acquisition method: users pinch the metal electrodes for 30 s while the device records a single-lead ECG. All HeartVoice VT signals in our study were collected via this handheld protocol. We apply the same preprocessing as for public datasets—each 30 s record is denoised, resampled to 200Hz, and segmented into 2 s windows—with a stride of 0.6 s for VT samples and 2 s for non-VT samples. For personalized adaptation (the pre-fine-tuning stage in MetaVA), we select 10 VT and 10 normal windows per patient, hold out 20 additional segments for validation, and reserve the remainder for testing. Hyperparameters mirror those used on public data, with the number of pre-fine-tuning iterations *iter* ∈{10,30,50} and update steps updates∈{1,3,5}.

**Fig 4:**
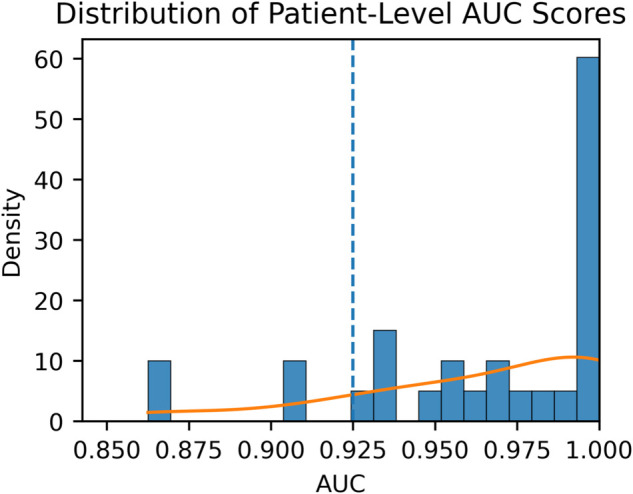
Patient-level AUC distribution on the HeartVoice VT cohort. Each bar shows the number of subjects whose personalised MetaVA model achieved the indicated AUC range; the orange KDE curve visualises the density, and the dashed vertical line marks the cohort mean ($\text{A}\bar{\text{U}}\text{C}=0.93$). The heavy concentration of bars to the right of 0.94 and the long tail peaking at 1.00 indicate that MetaVA consistently attains near-perfect discrimination for the vast majority of real-world patients.

Despite this minimal per-patient supervision, MetaVA maintains high discriminative performance on the HeartVoice cohort, achieving ROC-AUC 0.965, accuracy 0.931, and F1-score 0.937. These results closely match those on the VFDB test set (ROC-AUC 0.984, accuracy 0.960, F1-score 0.940) while offering greater clinical relevance by reflecting characteristics of contemporary patient data. Beyond aggregate metrics, Fig 4 shows the per-patient ROC-AUC distribution after individual fine-tuning: more than 80% of HeartVoice users exceed 0.94 ROC-AUC, with a substantial mode at 1.00, and the cohort-wide mean remains above 0.93. The tight, right-skewed distribution indicates that MetaVA generalizes effectively across diverse clinical signals; once meta-initialized, the model requires only a few labeled beats to attain near-perfect VT detection for most new patients. This validates both the effectiveness of our MAML-based pre-fine-tuning and the feasibility of learning under extremely limited, highly variable real-world conditions.

### Interpretation analysis

**Fig 5:**
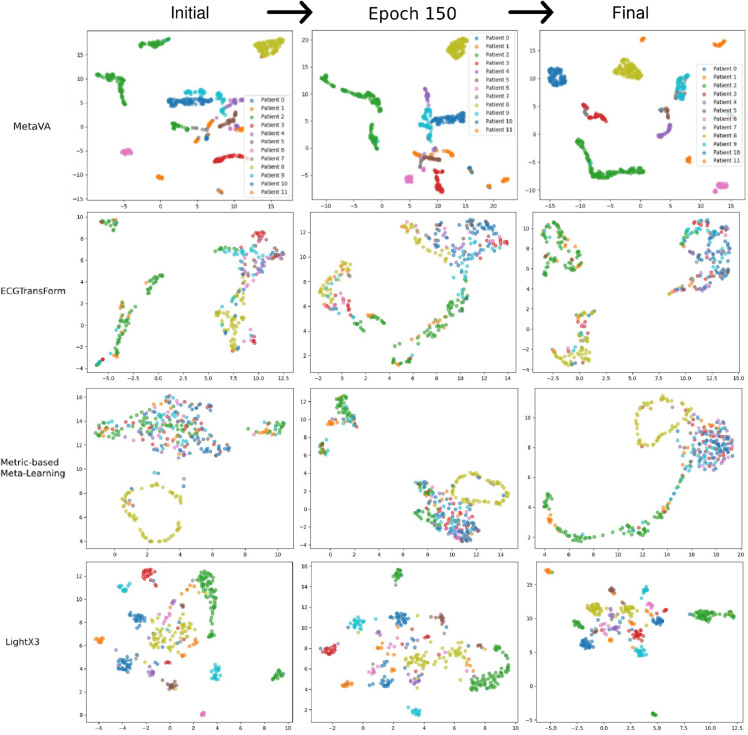
Embedding visualizations across training stages (arrows indicate chronology). The first row shows the embeddings produced by our MetaVA model: as training proceeds, VA segments from the same patient rapidly contract into tight, well-separated clusters, indicating that the meta-learner captures subject-specific morphology. Under identical settings, the three baselines yield embeddings that remain mixed, lacking patient-level separation. The contrast confirms that MetaVA learns more discriminative and personalised representations throughout optimisation.

To demonstrate MetaVA’s effectiveness in handling inter-subject variability, we visualized the embedding representations of VA segments from the VFDB test set. As shown in Fig 5, the top row (our MetaVA model) displays embeddings that form clear, patient-specific clusters, demonstrating how MetaVA both captures individual morphology and preserves separation between subjects. In contrast, the embeddings generated by the three state-of-the-art baseline models show substantial overlap between VA segments from different patients, with little to no distinct subject-wise clustering. This comparison highlights MetaVA’s superior capability in modeling inter-subject diversity. The observed clustering behavior can be attributed to MetaVA’s meta-learning strategy, which is optimizes for rapid adaptation at the subject level. This strategy enables the model to learn representations that generalize well across individuals while remaining sensitive to personal physiological variations.

## Discussion

In this work, we introduce MetaVA, a novel approach for detecting ventricular arrhythmias from ECGs that intend to address both inter-subject diversity and intra-subject variability. Specifically, our method uses a model-agnostic meta-learning (MAML) framework combined with a curriculum learning strategy during pre-training to effectively handle the differences among individuals (inter-subject diversity). We also introduce a novel pre-fine-tuning stage before the final fine-tuning, which enhances the model’s ability to adapt to each new subject’s unique ECG patterns and temporal changes (intra-subject variability). This two-stage learning strategy leads to superior performance in ventricular arrhythmia detection, with MetaVA outperforming both baseline and state-of-the-art methods across all evaluation metrics. Moreover, the MetaVA model is lightweight, enabling rapid personalization for new subjects and supporting deployment in low-resource settings. This underscores the practical applicability and clinical relevance of our approach.

Experimental results show that the MetaVA model outperforms traditional and state-of-the-art methods in ventricular arrhythmia (VA) detection, as seen in Table 9. This stems from integrating the CL-selector and pre-fine-tuning into the Model-Agnostic Meta-Learning (MAML) framework. Pre-training boosts generalization across individuals, while pre-fine-tuning ensures robust adaptation to new subjects and temporal changes within subjects.

Our analysis reveals the following key insights: (1)Meta-Learning with MAML: MAML effectively identifies highly adaptable initialization weights by treating each subject as a unique task, allowing robust generalization for inter-subject ECG variability and leading to improved VA detection. (2)CL-Selector: The CL-selector extends the "easy-to-hard" training paradigm by dynamically optimizing the task sampling process. This results in a smoother and more effective learning strategy, particularly for complex and noisy cases. (3)Pre-Fine-Tuning: Inspired by MAML, the pre-fine-tuning targets the intra-subject variability. It enhances efficient subject-level adaptation with minimal data, thereby enhancing generalization within individual patients.

Embedding visualizations (Fig 5) further validate MetaVA’s capability for effective personalization. In the embedding space learned by MetaVA, VA segments from the same subject form compact and well-separated clusters, in contrast to the mixed and overlapping distributions produced by traditional methods. This subject-specific clustering highlights MetaVA’s ability to capture individual morphological patterns, which is essential for robust performance across diverse ECG applications.

Most existing VA detection methods rely on either handcrafted feature extraction or large parameter-heavy models trained on extensive datasets [13,14,19,20]. Although recent personalization approaches [17,18] have accelerated adaptation, they remain constrained by their dependence on specific network achitectures. In contrast, MetaVA adopts a model-agnostic meta-learning framework that can be applied to a wide range of backbone achitectures. For instance, while MetaSleepLearner [41] targets rapid adaptation in the context of sleep staging, MetaVA extends this direction by incorporating both CL and pre-fine-tuning strategy tailored to arrhythmia detection. The integration of these components allows MetaVA to address both inter- and intra-subject variability more effectively. Furthermore, our method is complementary to architecture-focused improvements. Combining MetaVA with optimized network designs could lead to further gains in performance and efficiency.

MetaVA demonstrates strong generalization across subjects, and its performance could be further improved through several modifications:

(1) **Reptile substitution**: Replace the MAML update with a single-step Reptile update rule [42].
(2) **First-order approximations**: Retain the MAML structure but adopt lightweight first-order variants such as FOMAML or ANIL to reduce computational overhead [43,44].
(3) **Gradient sharing**: Introduce projection-based gradient-sharing modules to reuse shared descent directions across tasks and reduce adaptation latency [45].

Despite its empirical effectiveness, our method presents several limitations. While MAML supported by a solid theoretical foundation, both CL and pre-fine-tuning remain largely heuristic, lacking formal theoretical guarantees. Recent studies have also shown that anti-curriculum strategies [46] may offer comparable or even superior performance under certain conditions. Moreover, the computational cost of MAML’s second-order derivatives restricts its scalability to large networks. Finally, the effectiveness of our approach in handling real-world data with mislabeled entries remains uncertain. Clinically, false positives contribute to alarm fatigue and increased care burden; recent work in in-hospital ECG monitoring and remote/wearable programs highlights substantial rates of non-actionable or false alerts that can desensitize staff and provoke patient anxiety, potentially driving unnecessary utilization [47]; subject-specific fine-tuning may help balance sensitivity and specificity.

## Conclusion

In this study, we propose MetaVA, a VA detection framework designed to rapidly and accurately adapt to new individuals by transferring generalizable initialization parameters. Building upon the standard MAML framework, we enhance task selection through the integration of a curriculum learning (CL)-based task selector, and introduce a pre-fine-tuning stage that incorporates latent knowledge from meta-training into individual adaptation. Experiments are conducted on three public datasets (MITDB, CUDB and VFDB) and one real-world clinical dataset consisting of single-lead recordings (HeartVoice VT Clinical Data) using an advanced deep neural network architecture. After meta-training, the model is transferred to unseen individuals and fine-tuned using only 10 samples per class, achieving fast and effective personalization. Experimental results demonstrate that MetaVA consistently outperforms both conventional pre-training/fine-tuning strategies and selected baseline methods across all four evaluation metrics. In addition, embedding visualizations demonstrate that MetaVA effectively captures subject-specific morphological patterns, which is essential for addressing inter-individual variability in VA detection. Although some limitations remain, such as computational cost and sensitivity to annotation noise, the proposed approach shows strong potential for achieving both improved generalization and rapid adaptation in real-world clinical settings.

## References

[1] Hedman A, Hartikainen J, Vanninen E, Laitinen T, Jääskeläinen P, Laakso M, et al. Inducibility of life-threatening ventricular arrhythmias is related to maximum left ventricular thickness and clinical markers of sudden cardiac death in patients with hypertrophic cardiomyopathy attributable to the Asp175Asn mutation in the alpha-tropomyosin gene. J Mol Cell Cardiol. 2004;36(1):91–9. doi: 10.1016/j.yjmcc.2003.10.003 14734051

[2] Martin SS, Aday AW, Allen NB, Almarzooq ZI, Anderson CAM, Arora P, et al. 2025 heart disease and stroke statistics: A report of US and global data from the American Heart Association. Circulation. 2025;151(8):e41–660. doi: 10.1161/CIR.0000000000001303 39866113 PMC12256702

[3] Steinberg JS, Varma N, Cygankiewicz I, Aziz P, Balsam P, Baranchuk A, et al. 2017 ISHNE-HRS expert consensus statement on ambulatory ECG and external cardiac monitoring/telemetry. Heart Rhythm. 2017;14(7):e55–96. doi: 10.1016/j.hrthm.2017.03.038 28495301

[4] Cook DA, Oh S-Y, Pusic MV. Accuracy of physicians’ electrocardiogram interpretations: A systematic review and meta-analysis. JAMA Intern Med. 2020;180(11):1461–71. doi: 10.1001/jamainternmed.2020.3989 32986084 PMC7522782

[5] Ansari Y, Mourad O, Qaraqe K, Serpedin E. Deep learning for ECG Arrhythmia detection and classification: An overview of progress for period 2017 -2023. Front Physiol. 2023;14:1246746. doi: 10.3389/fphys.2023.1246746 37791347 PMC10542398

[6] Shah HA, Saeed F, Diyan M, Almujally NA, Kang J. ECG-TransCovNet: A hybrid transformer model for accurate arrhythmia detection using Electrocardiogram signals. CAAI Trans on Intel Tech. 2024. doi: 10.1049/cit2.12293

[7] Islam MS, Hasan KF, Sultana S, Uddin S, Lio’ P, Quinn JMW, et al. HARDC : A novel ECG-based heartbeat classification method to detect arrhythmia using hierarchical attention based dual structured RNN with dilated CNN. Neural Netw. 2023;162:271–87. doi: 10.1016/j.neunet.2023.03.004 36921434

[8] Zhou F, Fang D. Multimodal ECG heartbeat classification method based on a convolutional neural network embedded with FCA. Sci Rep. 2024;14(1):8804. doi: 10.1038/s41598-024-59311-0 38627498 PMC11639721

[9] Ding C, Yao T, Wu C, Ni J. Advances in deep learning for personalized ECG diagnostics: A systematic review addressing inter-patient variability and generalization constraints. Biosens Bioelectron. 2025;271:117073. doi: 10.1016/j.bios.2024.117073 39708490

[10] Carvalho M, Brás S. Addressing intra-subject variability in electrocardiogram-based biometric systems through a hybrid architecture. Biomed Signal Process Control. 2024;87:105465. doi: 10.1016/j.bspc.2023.105465

[11] Subba T, Chingtham T. Comparative analysis of machine learning algorithms with advanced feature extraction for ECG signal classification. IEEE Access. 2024;12:57727–40. doi: 10.1109/access.2024.3387041

[12] Xue J, Yu L. Applications of machine learning in ambulatory ECG. Hearts. 2021;2(4):472–94. doi: 10.3390/hearts2040037

[13] Acharya UR, Fujita H, Oh SL, Raghavendra U, Tan JH, Adam M, et al. Automated identification of shockable and non-shockable life-threatening ventricular arrhythmias using convolutional neural network. Future Gener Comput Syst. 2018;79:952–9. doi: 10.1016/j.future.2017.08.039

[14] Mathews SM, Kambhamettu C, Barner KE. A novel application of deep learning for single-lead ECG classification. Comput Biol Med. 2018;99:53–62. doi: 10.1016/j.compbiomed.2018.05.013 29886261

[15] Ribeiro AH, Ribeiro MH, Paixão GMM, Oliveira DM, Gomes PR, Canazart JA, et al. Automatic diagnosis of the 12-lead ECG using a deep neural network. Nat Commun. 2020;11(1):1760. doi: 10.1038/s41467-020-15432-4 32273514 PMC7145824

[16] Dong Y, Zhang M, Qiu L, Wang L, Yu Y. An arrhythmia classification model based on vision transformer with deformable attention. Micromachines (Basel). 2023;14(6):1155. doi: 10.3390/mi14061155 37374741 PMC10302689

[17] Jia Z, Wang Z, Hong F, Ping L, Shi Y, Hu J. Personalized deep learning for ventricular arrhythmias detection on medical IoT systems; 2020. https://doi.org/arXiv:2008.08060

[18] Fan L, Chen B, Zeng X, Zhou J, Zhang X. Knowledge-enhanced meta-transfer learning for few-shot ECG signal classification. Expert Syst Appl. 2025;263:125764. doi: 10.1016/j.eswa.2024.125764

[19] Hong S, Xiao C, Ma T, Li H, Sun J. MINA: Multilevel knowledge-guided attention for modeling electrocardiography signals. In: Proceedings of the twenty-eighth international joint conference on artificial intelligence; 2019. p. 5888–94. 10.24963/ijcai.2019/816

[20] Islam MR, Qaraqe M, Qaraqe K, Serpedin E. CAT-Net: Convolution, attention, and transformer based network for single-lead ECG arrhythmia classification. Biomed Signal Process Control. 2024;93:106211. doi: 10.1016/j.bspc.2024.106211

[21] Sakli N, Ghabri H, Soufiene BO, Almalki FA, Sakli H, Ali O, et al. ResNet-50 for 12-lead electrocardiogram automated diagnosis. Comput Intell Neurosci. 2022;2022:7617551. doi: 10.1155/2022/7617551 35528345 PMC9071921

[22] Vaid A, Jiang J, Sawant A, Lerakis S, Argulian E, Ahuja Y, et al. A foundational vision transformer improves diagnostic performance for electrocardiograms. NPJ Digit Med. 2023;6(1):108. doi: 10.1038/s41746-023-00840-9 37280346 PMC10242218

[23] An X, Shi S, Wang Q, Yu Y, Liu Q. Research on a lightweight arrhythmia classification model based on knowledge distillation for wearable single-Lead ECG monitoring systems. Sensors (Basel). 2024;24(24):7896. doi: 10.3390/s24247896 39771635 PMC11679805

[24] Chen Z, Yang D, Cui T, Li D, Liu H, Yang Y, et al. A novel imbalanced dataset mitigation method and ECG classification model based on combined 1D_CBAM-autoencoder and lightweight CNN model. Biomed Signal Process Control. 2024;87:105437. doi: 10.1016/j.bspc.2023.105437

[25] Rahman MM, Rivolta MW, Badilini F, Sassi R. A systematic survey of data augmentation of ECG signals for AI applications. Sensors (Basel). 2023;23(11):5237. doi: 10.3390/s23115237 37299964 PMC10256074

[26] Hospedales T, Antoniou A, Micaelli P, Storkey A. Meta-learning in neural networks: A survey. IEEE Trans Pattern Anal Mach Intell. 2022;44(9):5149–69. doi: 10.1109/TPAMI.2021.3079209 33974543

[27] Gharoun H, Momenifar F, Chen F, Gandomi AH. Meta-learning approaches for few-shot learning: A survey of recent advances. ACM Comput Surv. 2024;56(12):1–41. doi: 10.1145/3659943

[28] Finn C, Abbeel P, Levine S. Model-agnostic meta-learning for fast adaptation of deep networks. In: Proceedings of the 34th international conference on machine learning; 2017. p. 1126–35. https://proceedings.mlr.press/v70/finn17a.html

[29] Bengio Y, Louradour J, Collobert R, Weston J. Curriculum learning. In: Proceedings of the 26th annual international conference on machine learning; 2009. p. 41–8. 10.1145/1553374.1553380

[30] Glorot X, Bengio Y. Understanding the difficulty of training deep feedforward neural networks. In: Proceedings of the thirteenth international conference on artificial intelligence and statistics; 2010. p. 249–56. https://proceedings.mlr.press/v9/glorot10a.html

[31] El-Ghaish H, Eldele E. ECGTransForm: Empowering adaptive ECG arrhythmia classification framework with bidirectional transformer. Biomed Signal Process Control. 2024;89:105714. doi: 10.1016/j.bspc.2023.105714

[32] Radosavovic I, Kosaraju RP, Girshick R, He K, Dollár P. Designing network design spaces; 2020. https://doi.org/arXiv:2003.13678

[33] Xie S, Girshick R, Dollar P, Tu Z, He K. Aggregated residual transformations for deep neural networks. In: 2017 IEEE conference on computer vision and pattern recognition (CVPR), 2017. 10.1109/cvpr.2017.634

[34] Vaswani A, Shazeer N, Parmar N, Uszkoreit J, Jones L, Gomez AN, et al. Attention is all you need. Adv Neural Inform Process Syst. 2017. p. 5998–6008.

[35] PhysioNet. MIT-BIH arrhythmia database. PhysioNet. 2005. https://physionet.org/content/mitdb/1.0.0/

[36] PhysioNet. MIT-BIH malignant ventricular ectopy database; 1999. https://physionet.org/content/vfdb/1.0.0/

[37] PhysioNet. CU ventricular tachyarrhythmia database. PhysioNet; 2007. https://physionet.org/content/cudb/1.0.0/

[38] Sun S, Geng ZH, Zhang D. HeartVoice VT/non-VT single-lead ECG data. Zenodo. 2025. 10.5281/zenodo.16947288

[39] Liu Z, Chen Y, Zhang Y, Ran S, Cheng C, Yang G. Diagnosis of arrhythmias with few abnormal ECG samples using metric-based meta learning. Comput Biol Med. 2023;153:106465. doi: 10.1016/j.compbiomed.2022.106465 36610213

[40] Le KH, Pham HH, Nguyen TBT, Nguyen TA, Thanh TN, Do CD. LightX3ECG: A lightweight and eXplainable deep learning system for 3-lead electrocardiogram classification. Biomed Signal Process Control. 2023;85:104963. doi: 10.1016/j.bspc.2023.104963

[41] Banluesombatkul N, Ouppaphan P, Leelaarporn P, Lakhan P, Chaitusaney B, Jaimchariyatam N, et al. MetaSleepLearner: A pilot study on fast adaptation of bio-signals-based sleep stage classifier to new individual subject using meta-learning. IEEE J Biomed Health Inform. 2021;25(6):1949–63. doi: 10.1109/JBHI.2020.3037693 33180737

[42] Nichol A, Achiam J, Schulman J. On first-order meta-learning algorithms; 2018. https://arxiv.org/abs/1803.02999

[43] Chayti EM, Jaggi M. A new first-order meta-learning algorithm with convergence guarantees; 2024. https://doi.org/arXiv:2409.03682

[44] Yüksel OK, Flammarion N. First-order ANIL provably learns representations despite overparametrization; 2024. https://doi.org/arXiv:2303.01335

[45] Chang HL. Accelerating meta-learning by sharing gradients; 2023. https://doi.org/arXiv:2312.08398

[46] Liu F, Zhang T, Zhang C, Liu L, Wang L, Liu B. A review of the evaluation system for curriculum learning. Electronics. 2023;12(7):1676. doi: 10.3390/electronics12071676

[47] Cosentino N, Zhang X, Farrar EJ, Yapici HO, Coffeng R, Vaananen H, et al. Performance comparison of 6 in-hospital patient monitoring systems in the detection and alarm of ventricular cardiac arrhythmias. Cardiovasc Digit Health J. 2024;5(2):70–7. doi: 10.1016/j.cvdhj.2024.02.002 38765622 PMC11096657

